# Acupuncture for treating tic disorders in children: A protocol for systematic review and meta-analysis

**DOI:** 10.1097/MD.0000000000024860

**Published:** 2021-03-26

**Authors:** Qianfang Fu, Xilian Zhang, Haihong Yan, Jiabao Xu, Hui Liu, Libin Yang, Shuyi Zhao, Ping Rong, Rong Ma

**Affiliations:** a Department of Pediatrics, First Teaching Hospital of Tianjin University of Traditional Chinese Medicine, Xiqing; b National Clinical Research Center for Chinese Medicine Acupuncture and Moxibustion, Xiqing; c School of Traditional Chinese Medicine, Tianjin University of Traditional Chinese Medicine, Jinghai, Tianjin, China.

**Keywords:** acupuncture, children, meta-analysis, protocol, systematic review, tic disorders

## Abstract

**Background:**

Tic disorders (TDs) are a group of neurodevelopmental disorders in children, while pharmacotherapy is often associated with various side effects and has limited clinical effects for some patients, thus significantly affecting patients’ quality of life. Studies have found acupuncture shows certain advantages in the treatment of TDs. However, there is no high level of evidence evaluating the effectiveness and safety of acupuncture for children with TDs.

**Methods:**

Each data of acupuncture for treating TDs will be searched. We will search for related English and Chinese databases. The time is limited from inception until November 2020. The primary outcome is the reduction rate (amount) of tic severity using related scales or methods, and the secondary outcomes include recurrence rate and adverse events. The risk of bias will be assessed, and the RevMan5.3 and Stata14.0 will be performed for meta-analysis. Finally, we will assess the level of the resulting evidence.

**Results:**

The results of the study will synthesize the current evidence and be published in peer-reviewed journals.

**Conclusions:**

This research aims to provide convincing evidence of the effectiveness and safety of acupuncture for treating TDs in children.

**INPLASY registration number:**

INPLASY2020110050.

## 1 Introduction

Tic disorders (TDs) are characterized by rapid, sudden, nonrhythmic, and motor and/or phonic movements that are repetitive and out of context, with onset mostly in childhood.^[1]^ Based on the type of tic, tic duration, age of beginning, and absence of other causes, TDs are classified into 3 diagnostic categories: transient tic disorder, persistent vocal or motor tic disorder, and Tourette's syndrome (TS) with prevalence rates of 1.7%, 1.2%, and 0.3%, respectively in China.^[2]^ In addition to tics, about half of the children with TDs and most individuals (an estimated 86%–90%) with TS, which denotes the most severe form of TDs, have more than 1 coexisting neurodevelopmental problem.^[3–5]^ These problems are known as attention deficit hyperactivity disorder (ADHD), disruptive behaviors, self-injurious behavior, mood disorders, etc.^[6,7]^ TDs and these co-occurring comorbidities are associated with functional impairment, emotional impairment, and educational impairment for children.^[8]^

The first-line agents for patients requiring pharmacological treatment remain dopaminergic receptor blockades and alpha-adrenergic agonists.^[9]^ However, pharmacological treatments have limited clinical effects for some patients and have been limited by various side effects, including extrapyramidal symptoms, sedation, sleep disturbance, and metabolic disorders.^[10]^ Besides, nonpharmacologic treatment, such as cognitive-behavioral therapy, is not easily available in China currently.^[11]^ Therefore, effective and safe treatments for TDs are in urgent need. In recent years, clinical studies have found acupuncture shows certain advantages in the treatment of TDs and is well tolerated.^[12]^ As said by the theory of traditional Chinese medicine (TCM), acupuncture can adjust the balance of “Qi and blood,” coordinate thoroughfare, and dredge “meridian and collateral” by stimulating acupoints.^[13]^ Besides, the study found acupuncture can promote blood infusion in regions of the brain that are dysfunctional.^[14]^

To date, we only sought out 1 published Chinese meta-analysis on acupuncture for TDs in children, which was published on May 15, 2017. In this study, most of the studies included were small sample sizes thus influencing the findings.^[15]^ After preliminary searches, we found the number of trials of acupuncture for TDs is increasing in recent years. With a more thorough retrieval strategy and more included randomized controlled trials (RCTs), our research aims to provide convincing conclusions of the effectiveness and safety of acupuncture for TDs in children.

## 2 Methods

### 2.1 Study registration

We have registered at INPLASY (registration number: INPLASY2020110050; https://inplasy.com/inplasy-2020-11-0050/).

### 2.2 Inclusion criteria

#### 2.2.1 Types of studies

All RCTs evaluating the effectiveness and safety of acupuncture treatment for TDs will be included without publication type restriction. The language will be limited to English and Chinese. Conference papers, protocol, case report, review, animal study, comments, and supplementary issues will be excluded.

#### 2.2.2 Types of patients

Patients who met the definitions of TDs will be included. And the inclusion criteria required patients enrolled to be younger than 18 years.

#### 2.2.3 Types of interventions and controls

Any form of acupuncture treatment, such as body acupuncture, auricular acupuncture, scalp acupuncture, dermal needle, and electroacupuncture will be included, regardless of frequency, intensity, and duration. Point injection, acupoint application, acupressure, laser acupuncture, cupping, and moxibustion considered to be another part of TCM will be eliminated. Besides, acupuncture combined with other effective interventions will be included. Placebo, no cure, false acupuncture, and other therapies (e.g., drug treatment and other active therapies) will be included as control interventions. However, studies comparing the effectiveness of different forms of acupuncture will be excluded.

#### 2.2.4 Types of outcomes

The primary outcome is the reduction rate (amount) of tic severity measured by related scales. In order of preference, the scales include the Yale Global Tic Severity Scale, Tourette Syndrome Global Scale, The Gilles de la Tourette Syndrome-Quality of Life Scale, Shapiro Tourette Syndrome Severity Scale, and other scales or methods. The secondary outcomes include recurrence rate and adverse events.

#### 2.2.5 Electronic searches

Five English databases (PubMed, EMBASE, Web of Science, International Clinical Trials Registry Platform, Cochrane Library) and 4 Chinese databases (Wan-fang database, China National Knowledge Infrastructure, Chinese Biomedical Literature Database, VIP Database) will be searched. The time is limited from inception until November 2020. In addition, the Chinese Clinical Trial Registry Centers and Grey Literature will also be searched. Authors will be contacted for further information if essential data are missing. The searching strategy of PubMed is summarized in Table 1.

**Table 1:** Search strategy used in PubMed database.

Number	Search terms
1	Randomized controlled trial
2	Controlled clinical trial
3	Randomized
4	Randomly
5	Randomised
6	Trial
7	or 1–6
8	Tic Disorders
9	Tic Disorder
10	Transient Tic Disorder
11	Chronic Motor or Vocal Tic Disorder
12	Post-Traumatic Tic Disorder
13	Childhood Tic Disorder
14	or 8–13
15	Acupuncture therapy
16	Acupuncture
17	Acupoint
18	Electroacupuncture
19	Electro-acupuncture
20	Fire needle
21	dermal needle
22	Scalp acupuncture points
23	Auricular points
24	or 15–23
25	7 and 14 and 24

#### 2.2.6 Searching other resources

The following literature founded earlier than the database in China, such as “*Chinese Journal of Rehabilitation Medicine Journal*,” “*Chinese Acupuncture and Moxibustion*,” and “*Acupuncture Research”* will also be searched.

### 2.3 Data management and analysis

#### 2.3.1 Studies selection

**Figure 1:**
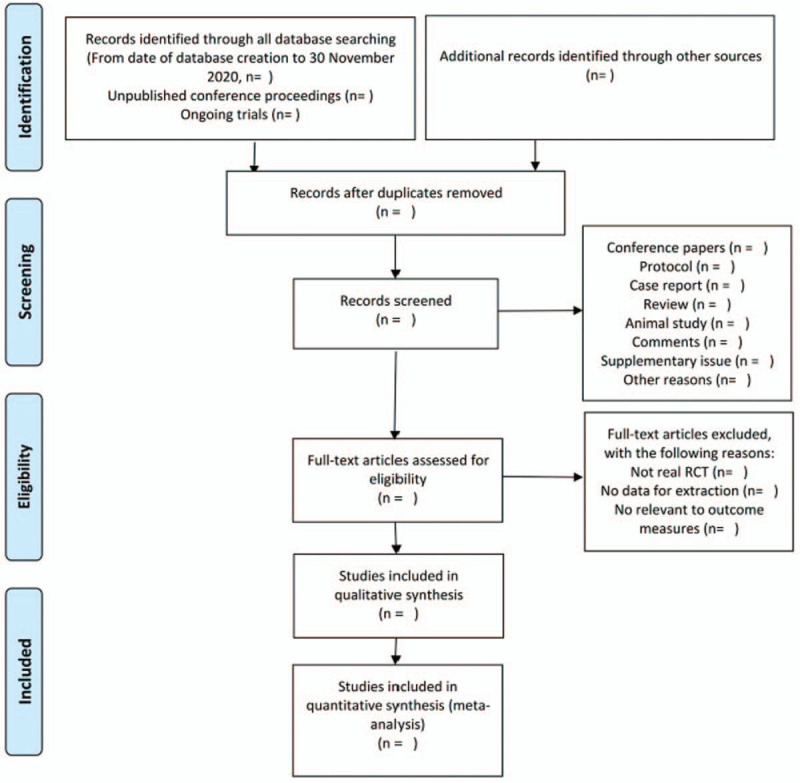
Flow diagram of studies identified.

Two researchers (QFF, XLZ) will receive professional training for this study and we will evaluate the consistency between the 2 reviewers. All electronic citations retrieved from the database using the search strategy mentioned above will be imported into Endnote X9 and duplicate studies will be excluded. First of all, QFF and XLZ will screen the titles and abstracts independently to filter out the literature that did not accord with the pre-established standards. Secondly, carefully reading the remaining documents and deciding whether to include them or not. Eventually, discussing the results together. If they have any disagreement, the 3rd party (PR or RM) needs to make the final decision. The screening flow chart is presented in the PRISMA flow diagram (Fig. 1).

#### 2.3.2 Data extraction

Two investigators (HHY and JBX) will input the data independently using the data form uniformly developed by the researcher. The information of each selected trial includes basic information of studies (article title, author details, time of publication research, sources of funds), sample size, grouping method, patients’ gender, age, diagnostic criteria, severity, acupuncture brand, acupuncture point, the quantity of stimulus information, efficacy evaluation, adverse effects, and recurrence rate.

#### 2.3.3 Risk of bias assessment

Two authors (HL and LBY) will assess the risk of bias independently adopting the Cochrane Collaboration's risk of bias tool. For each item, the risk of bias will be rated as: “low,” “unclear,” or “high.” Disagreements will be solved first by discussion and then by seeking the judgment of a third author (PR).

#### 2.3.4 Statistical analysis

We will analyze the data using the RevMan V.5.3.0. With 95% confidence intervals, a risk ratio (RR) will be analyzed for dichotomous data, and standardized mean difference (SMD) or mean difference (MD) will be analyzed for the continuous variable outcomes, depending on whether different scales are used to measure an outcome. A *P* value <.05 will be defined as statistically significant. Two authors (QFF and SYZ) will perform this work independently to reduce inaccuracy.

#### 2.3.5 Heterogeneity assessment

*P* values and Chi-Squared test will be calculated depending on the value of *I*^2^. When *I*^2^ ≥ 50%, considerable heterogeneity will be confirmed, and we will choose the random effect model. If not, a fixed-effect model. After removing the major or unacceptable sources of heterogeneity, a meta-analysis will be conducted. In addition, if the source of heterogeneity cannot be found, a systematic review will be performed.

#### 2.3.6 Subgroup analysis

According to the differences in acupuncture methods, category of disease, treatment frequencies, control group, and follow-up duration, we will perform the subgroup analysis.

#### 2.3.7 Sensitivity analysis

Sensitivity analysis will be performed according to the following criteria: the quality of studies, sample size, missing data, statistical model, and heterogeneity qualities.

#### 2.3.8 Publication bias

When included studies are more than 10, a funnel plot or Egger test will be performed.^[16]^

#### 2.3.9 Quality assessment

Considering the following aspects: consistency, bias risk, precision, indirectness, and publication bias, we will apply the Grading of Recommendation Assessment, Development and Evaluation approach to assess the quality of evidence.^[17]^

### 2.4 Ethics and dissemination

The data will be collected from published literature, so no ethical approval is inquired. The results of the study will be published in peer-reviewed journals. If the protocol is modified, the information will be described in the final report.

## 3 Discussion

TDs and their co-occurring comorbidities bring children impairment and place a burden on families and society, while pharmacological treatments are often associated with significant side-effects. Besides, nonpharmacologic therapy is not easily available for patients in China. Therefore, complementary alternative medicine, such as acupuncture and TCM, have become available to patients with TDs and their caregivers.^[18]^ In China, guidelines of TCM also recommend the use of acupuncture for TDs.^[19]^ However, robust data about the clinical effects and safety of acupuncture for treating children with TDs is lacking and previous studies are limited by small sample sizes and often ignore the potential side effects of acupuncture.^[20]^ In recent years, clinical RCTs of acupuncture for treating patients with TDs are increasing. With a more thorough retrieval strategy and more included RCTs, we hope this study can provide convincing clinical evidence and clinical decision scheme for clinicians.

## Author contributions

**Data curation:** Qianfang Fu, Xilian Zhang, Haihong Yan, Jiabao Xu.

**Formal analysis:** Qianfang Fu, Hui Liu, Libin Yang, Shuyi Zhao.

**Funding acquisition:** Ping Rong.

**Project administration:** Ping Rong, Rong Ma.

**Supervision:** Ping Rong, Rong Ma.

**Validation:** Ping Rong, Rong Ma.

**Writing – original draft:** Qianfang Fu, Xilian Zhang.

**Writing – review & editing:** Qianfang Fu, Xilian Zhang.
